# Dynamic regulation of *Arabidopsis* β-AMYLASE1 by glutathione and thioredoxins affects starch in guard cells

**DOI:** 10.1093/plphys/kiaf344

**Published:** 2025-08-01

**Authors:** Libero Gurrieri, Anna Clara Capuzzi, Stefanie J Müller-Schüssele, Paolo Trost, Francesca Sparla

**Affiliations:** Department of Pharmacy and Biotechnology, University of Bologna, 40126 Bologna, Italy; Department of Pharmacy and Biotechnology, University of Bologna, 40126 Bologna, Italy; Department of Biology, University of Kaiserslautern-Landau, 63663 Kaiserslautern, Germany; Department of Pharmacy and Biotechnology, University of Bologna, 40126 Bologna, Italy; Department of Pharmacy and Biotechnology, University of Bologna, 40126 Bologna, Italy

## Abstract

Guard cells control the opening and closure of stomatal pores in response to internal and external stimuli, ensuring gas exchange in plants. In *Arabidopsis* (*Arabidopsis thaliana*), β-AMYLASE1 (BAM1), assisted by α-AMYLASE3, begins degrading starch at dawn in guard cells to promote stomatal opening. Both enzymes are controlled by reversible disulfide bond formation, which decreases their activity. In the present study, we investigated the sensitivity of BAM1 to other redox-dependent post-translational modifications (PTM) both in vitro and in vivo. In vitro, H_2_O_2_ reversibly inactivates BAM1 and, in the presence of glutathione (GSH), induces *S*-glutathionylation of BAM1. Glutathionylated BAM1 is active and transiently protected from H_2_O_2_ inhibition. However, the glutathionylated state of BAM1 has limited stability and can be slowly resolved by a second cysteine with the formation of the intramolecular disulfide bond that inhibits BAM1 activity. Thioredoxin *f* can fully revert the inhibition by reducing the disulfide to a dithiol. In vivo, *Arabidopsis* mutants with lower plastidial GSH reductase activity, and consequently modified GSH homeostasis, showed higher BAM1 activity, lower starch levels in guard cells, and altered stomata aperture, indicating that GSH redox potential impacts stomatal physiology, possibly through BAM1. Moreover, plastidial BAM1 presents a prime example for the role of glutathionylation functioning as a transiently protective PTM, interfering with the formation of inhibitory disulfide bonds. This example illustrates how transitions between protein cysteinyl thiol PTMs can orchestrate dynamic responses involving several redox systems.

## Teaser

The redox homeostasis of GSH regulates starch metabolism in guard cells by influencing the activation state of the β-AMYLASE1, a key enzyme in guard cell starch degradation.

## Introduction

Metabolic regulation occurs at different levels, including transcriptional and translational control as well as post-translational protein modifications (PTMs). PTMs provide a rapid way to regulate enzyme activity in response to internal and environmental stimuli. Thiol-based redox modifications link reactive oxygen species (ROS)/reactive nitrogen species signaling, the availability of reducing power, and protein function (Noctor et al. 2018). In the case of primary carbon metabolism, the reversible formation of disulfide bonds enables the regulation of Calvin–Benson–Bassham cycle in response to light/dark conditions (Michelet et al. 2013; Gurrieri et al. 2021, 2024). This regulation involves several redox-active proteins including thioredoxins (TRXs), NADPH-thioredoxin reductase (NTRC), and 2-Cys peroxiredoxins, whose interplay synchronizes the light-harvesting phase with carbon-fixing reactions of photosynthesis (Cejudo et al. 2021; Yoshida and Hisabori 2023).

Redox regulation also affects starch metabolism (Santelia et al. 2015; Skryhan et al. 2018). Indeed, the activity of key enzymes involved in starch synthesis, e.g. ADP-glucose pyrophosphorylase (Ballicora et al. 2000), and degradation, e.g. glucan water dikinase (Mikkelsen et al. 2005), and several amylases (Sparla et al. 2006; Seung et al. 2013; Storm et al. 2018) are redox-regulated.

The first amylase to be identified as a target of reductive activation was β-AMYLASE1 (BAM1) from *Arabidopsis thaliana* (Sparla et al. 2006). BAM1 is inhibited by an intramolecular disulfide bond formation that can be reduced by TRXs or NTRC (Sparla et al. 2006; Valerio et al. 2011). From a physiological point of view, the TRX-dependent activation of BAM1 makes the enzyme active in the presence of light, which is a counterintuitive feature for an enzyme involved in transitory starch degradation (Lloyd et al. 2005). Diurnal starch breakdown occurs both in mesophyll (Zanella et al. 2016; Fernandez et al. 2017; Ishihara et al. 2022) and guard cells (Horrer et al. 2016; Flütsch et al. 2020). BAM1 is present in guard cells and is involved in the drought stress response, which requires diurnal starch degradation (Valerio et al. 2011; Zanella et al. 2016).

Starch in guard cells acts as a specialized carbon source for stomatal movements. Glucose molecules derived from diurnal starch degradation are converted into malate, which is accumulated in the vacuole, thereby contributing to the change in osmotic pressure driving the stomatal opening (Dittrich and Raschke 1977; Santelia and Lawson 2016; Daloso et al. 2017). Specifically, starch granules begin to be degraded in the last 3 h of the night, while synthesis resumes after a few hours of light (Horrer et al. 2016; Flütsch et al. 2022 ), raising questions on how starch metabolism might be controlled in these specialized cells.

Key players of starch degradation in stomata include BAM1, which is responsible for 80% of the degrading activity, and α-AMYLASE3 (AMY3; Horrer et al. 2016; Santelia and Lunn 2017), both regulated by the TRX system (Sparla et al. 2006; Seung et al. 2013 ). Notably, AMY3 activity can also be inhibited by glutathionylation (Gurrieri et al. 2019), a redox PTM known to play several roles in chloroplasts, including the regulation of protein function and the protection of thiols from over-oxidation during oxidative stress (Zaffagnini et al. 2012a , 2012b; Corpas et al. 2022).

Glutathione (GSH) is the major low-molecular-weight thiol in the cell and, together with ascorbate, is responsible for buffering redox changes and maintaining redox homeostasis in plants (Foyer and Noctor 2011; Deponte 2017). GSH can form a mixed disulfide with cysteine residues leading to protein *S*-glutathionylation (Zaffagnini et al. 2019; Yu et al. 2020). Glutathionylation can be induced by H_2_O_2_ that oxidizes deprotonated cysteines to sulfenic acids (–SOH). The sulfenic acid form of cysteines can then be rapidly glutathionylated nonenzymatically by GSH (Zaffagnini et al. 2012a, 2012b). Alternatively, glutathionylation of reduced cysteine residues can result from nonenzymatic equilibration with oxidized GSH or via Class I glutaredoxins (GRXs) acting as catalysts (Deponte 2017; Bohle et al. 2024).

Considering the important role of starch in guard cell physiology, and that of H_2_O_2_ in the regulation of stomatal movements (Rodrigues and Shan 2022; Lemonnier and Lawson 2024), we decided to study the effect of H_2_O_2_, alone and in the presence of GSH, on BAM1 activity. We show that H_2_O_2_-mediated *S*-glutathionylation of BAM1 is a transient modification that does not affect enzyme activity per se, as observed by Storm et al. (2018) for *S*-nitrosoglutathione-mediated *S*-glutathionylation, but it rather slows down the H_2_O_2_-dependent inactivation of BAM1. Only after prolonged incubation, the glutathionylation of BAM1 turns into an intramolecular disulfide that inhibits BAM1 activity. Moreover, plants with reduced amounts of glutathione reductase 2 (GR2) and thus higher oxidized glutathione (GSSG) concentration in vivo show higher BAM1 activity, lower starch content, and reduced stomatal opening than wild-type plants. Taken together, our results reveal that the stromal GSH redox state impacts guard cell functionality presumably via BAM1 regulation.

## Results

### Hydrogen peroxide reversibly inhibits BAM1 activity

**Figure 1.**
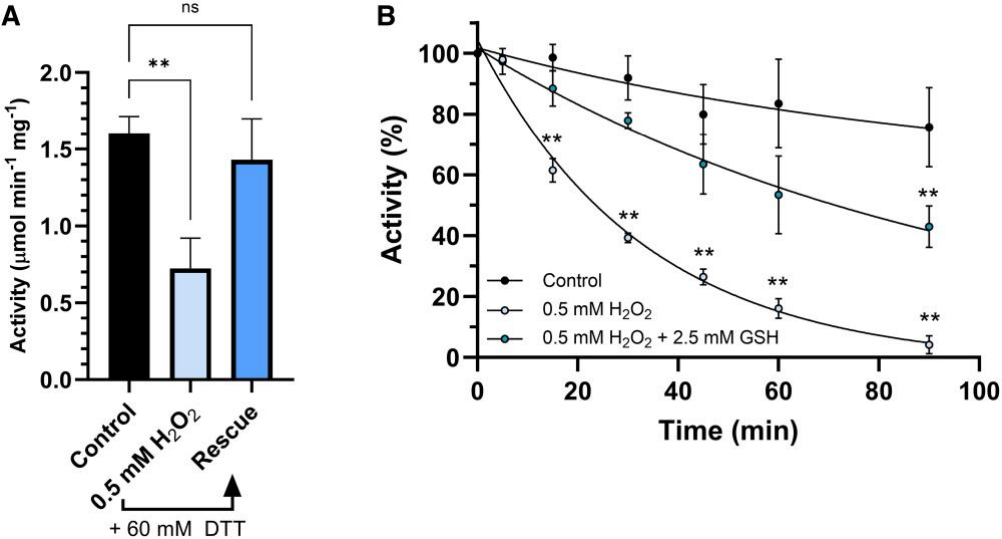
BAM1 sensitivity to H_2_O_2_ and protective effect of GSH. **A)** Inactivation of BAM1 after 1 h incubation with 0.5 mm H_2_O_2_. The control sample was incubated for 60 min in the absence of H_2_O_2_. Rescue of BAM1 activity was recorded in response to an additional 30 min incubation with DTT. **B)** Inactivation kinetics of BAM1 incubated with 0.5 mm H_2_O_2_ with or without 2.5 mm GSH (reduced GSH). The control sample has been incubated in the absence of any reagent. All experiments were carried out in triplicate; error bars show standard deviation. Data were analyzed with Student's *t*-test and compared to the control sample; **, *P* < 0.01; ns, not significant.

BAM1 harbors 8 cysteines, at least 2 of which (Cys32 and Cys470), are involved in redox regulation through the formation of a disulfide bond that inhibits enzyme activity (Sparla et al. 2006). We tested the in vitro sensitivity of BAM1 cysteines to oxidants by incubating purified BAM1 in the presence of 0.5 mm H_2_O_2_ for 1 h. The activity decreased by 60% (Fig. 1A). The subsequent incubation with 60 mm DTT completely restored BAM1 activity (Fig. 1A), suggesting that H_2_O_2_ might have caused the formation of an inhibitory disulfide bond that DTT could fully reduce, thereby activating the enzyme.

H_2_O_2_-induced disulfide bond formation typically occurs in 2 steps: sulfenylation of an acidic cysteine by H_2_O_2_ and nucleophilic attack on the sulfenyl group (–SOH) by a second cysteine thiol (Paulsen and Carroll 2013). Thus, disulfide bond formation may protect the sulfenylated cysteine from further oxidation by H_2_O_2_ to sulfinic (SO_2_H)/sulfonic (SO_3_H) groups, which cannot be reduced back to the original thiol form.

Similarly, sulfenylated cysteines could also be protected by reacting with the cysteinyl thiol group of GSH to form a mixed disulfide. Therefore, the effect of H_2_O_2_ on BAM1 activity was also monitored in the presence of GSH over time (Fig. 1B). Indeed, the presence of GSH slowed down the H_2_O_2_-dependent inhibition of BAM1, indicating that GSH could limit the formation of the inhibitory intramolecular disulfide, likely through a fast reaction with the sulfenic cysteine of BAM1.

### BAM1 is a target of glutathionylation

**Figure 2.**
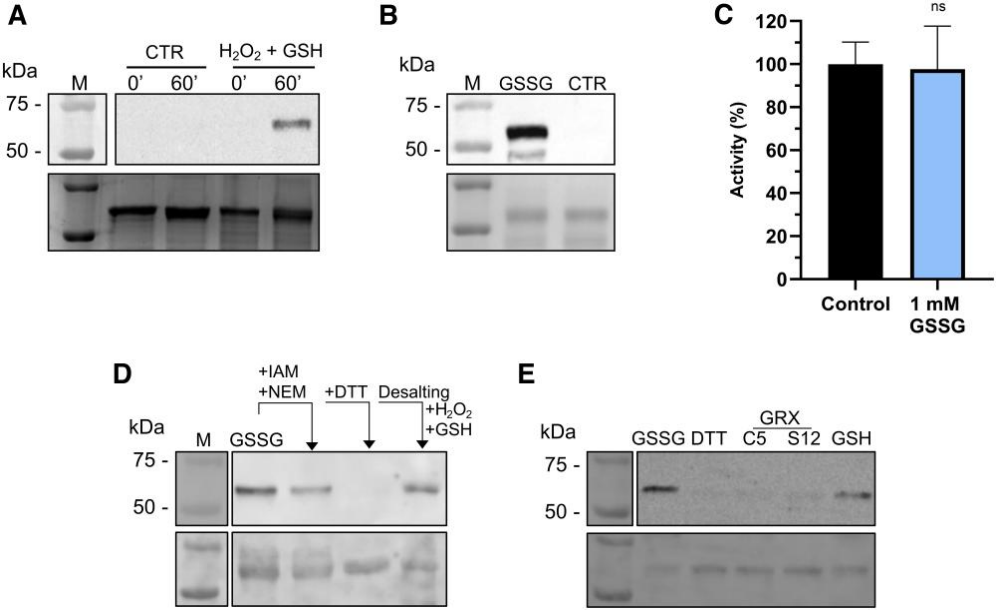
Glutathionylation of BAM1 has no effect on catalysis. **A)** Upper panel: Western blot analysis with α-GSH antibodies on BAM1 samples untreated (CTR) and treated with 0.5 mm H_2_O_2_ and 2.5 mm GSH (reduced GSH). Samples were analyzed at 0 min and after 60 min of incubation. Lower panel: Coomassie staining of the same samples. **B)** Upper panel: Western blot analysis with α-GSH antibodies on BAM1 samples untreated (CTR) and treated with 1 mm GSSG (oxidized GSH). Samples were analyzed after 60 min of incubation. Lower panel: Coomassie staining of analogous samples. **C)** BAM1 activity was measured in untreated (Control) and treated sample (1 mm GSSG) after 1 h of incubation. Data were analyzed with Student's *t*-test and compared to the control sample; ns, not significant (*P* < 0.05). The experiment was carried out in triplicate; error bars show standard deviation. **D)** Upper panel: Western blot analysis with α-GSH antibodies on BAM1 pre-treated with 1 mm GSSG. The remaining free cysteine thiols were blocked with alkylating agents (20 mm IAM; and 20 mm NEM), the sample was diluted and glutathionylation has been removed with 20 mm DTT. All the reagents were removed by desalting and the protein treated with 0.5 mm H_2_O_2_ and 2.5 mm GSH. The signal observed in this latter sample indicates that the same cysteine can be a target of both glutathionylating treatments (GSSG or H_2_O_2_ and GSH). Lower panel: Ponceau staining of the membrane after protein transfer. **E)** Deglutathionylation of BAM1 assayed by Western blot analysis using α-GSH antibodies. Upper panel: BAM1 was treated with 1 mm GSSG (GSSG lane), desalted, and then incubated with 0.5 mm DTT, 2 mm GSH alone or in the presence of 1 *µ*M GRX C5 or GRX S12. Lower panel: Ponceau staining of the membrane after protein transfer. A and E: The M lane images were acquired as colorimetric images by the same imaging system used to detect the chemiluminescence signal.

The possibility that GSH protected BAM1 from H_2_O_2_-induced inhibition by forming a mixed disulfide was investigated by Western blot analysis using α-GSH antibodies. For this purpose, BAM1 was incubated either with H_2_O_2_ plus GSH or with GSSG. Western blot analysis of both treatments revealed strong glutathionylation signals after 60 min of incubation (Fig. 2, A and B).

The incubation with GSSG helped disentangle the direct effect of H_2_O_2_ from those of glutathionylation. In fact, the activity of glutathionylated BAM1 after GSSG incubation was not inhibited compared to the control (Fig. 2C), in contrast with BAM1 incubated with GSH and H_2_O_2_ (Fig. 1B). This result suggests that either (i) treatments with GSSG or GSH and H_2_O_2_ do not target the same cysteine and therefore have different effects on BAM1 activity or (ii) glutathionylation does not impair BAM1 catalysis, and the partial inhibition observed after GSH and H_2_O_2_ treatment (Fig. 1B) depends on the formation of a disulfide induced by H_2_O_2_.

To demonstrate that glutathionylation affects the same site(s), the glutathionylation signal was tracked using α-GSH antibodies in Western blot analysis. Recombinant BAM1 underwent glutathionylation upon incubation with GSSG (Fig. 2D, second lane), after which the unmodified cysteines were blocked using the alkylating agents. iodoacetamide (IAM) and *N*-ethylmaleimide (NEM), (Fig. 2D, third lane). The sample was then diluted, and glutathionylation was removed by adding DTT (Fig. 2D, fourth lane). After the removal of reagents by desalting, BAM1 protein, with only the cysteine(s) previously targeted by GSSG now exposed in the thiol state, was treated with H₂O₂ and GSH, resulting in a new glutathionylation signal (Fig. 2D, fifth lane). This experiment revealed that the same cysteine(s) can be targeted by 2 different glutathionylation reactions. This finding excludes our first hypothesis and supports the second: the inhibition observed after H_2_O_2_ and GSH treatment could be due to H_2_O_2_ rather than glutathionylation of a different cysteine than the one modified by GSSG.

Considering that in vivo protein glutathionylation depends on GSH redox steady state in both nonenzymatic- and GRX-dependent reactions, we tested the regulation of BAM1 glutathionylation in vitro. For this purpose, glutathionylated BAM1 was incubated for 1 h with GRX C5 or GRX S12 in the presence of GSH (Fig. 2E). Control incubations were also performed with GSH and DTT (Fig. 2E). All treatments affected the glutathionylation of BAM1, especially GRXs and DTT, whereas GSH alone removed the GSH only partially.

### Spontaneous loss of GSH leads to inhibition of BAM1 by intramolecular disulfide formation

**Figure 3.**
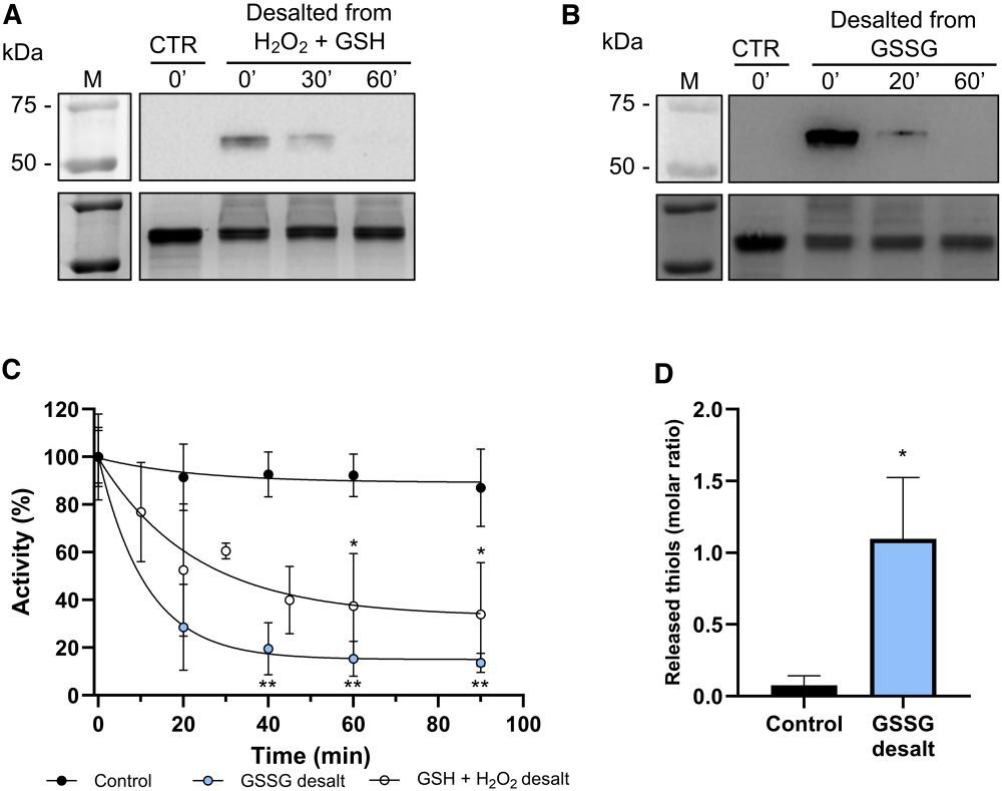
Spontaneous loss of GSH leads to BAM1 inhibition. **A)** Upper panel: BAM1 samples were incubated for 1 h with 0.5 mm H_2_O_2_ and 2.5 mm GSH or with an equal volume of buffer (CTR). After incubation both samples were desalted in 100 mm Tricine–NaOH pH 7.9 and at indicated time points, the enzyme activity were analyzed by Western blot analysis using α-GSH antibodies. Lower panel: Coomassie staining of the same samples. **B)** Upper panel: BAM1 samples were incubated for 1 h with 1 mm GSSG or with an equal volume of buffer (CTR). After incubation, both samples were desalted in 100 mm Tricine–NaOH pH 7.9 and at indicated time points, the enzyme activity were analyzed by Western blot analysis using α-GSH antibodies. Lower panel: Coomassie staining of the same samples. **C)** BAM1 activity measured on samples shown in A and B; the activity of every sample is expressed as percentage of the activity at 0 min after the desalting. **D)** Thiols were released from untreated (Control) and GSSG-treated BAM1 (as in B). Control and treated samples were desalted after 1 h and incubated for 90 min before measuring the released GSH in the flow-through of the ultrafiltered BAM1 samples. All experiments were carried out in triplicate; error bars show standard deviation. Data were analyzed with Student's *t*-test and compared to the untreated sample; **, *P* < 0.01; *, *P* < 0.05.

To further characterize the behavior of glutathionylated BAM1, enzyme preparations were desalted after 1 h of incubation to remove excess reagents (H_2_O_2_ and GSH, GSSG, or buffer). After desalting, both the glutathionylation state (Fig. 3, A and B) and enzyme activity (Fig. 3C) were monitored at different time points.

Western blot analyses revealed that glutathionylation signals disappeared over time (Fig. 3, A and B) and were no longer visible at 60 min after desalting. Activity assays on the same samples showed a decrease in BAM1 activity that corresponds to the loss of the glutathionylation signal (Fig. 3C). The lowest activities were recorded in samples that showed no glutathionylation signals (Fig. 3, A to C). Compared to control samples (Fig. 3C, black circle), both BAM1 samples pretreated with GSH plus H_2_O_2_ (Fig. 3C, white circle) and samples pretreated with GSSG (Fig. 3C, light blue circle) started losing activity after desalting, reaching a plateau at 15% to 25% of the initial activity within 40 to 60 min.

Given that BAM1 is inhibited by a regulatory disulfide bridge (Sparla et al. 2006; Valerio et al. 2011), the presence of soluble GSH in the medium was quantified when BAM1 reached its lowest activity (i.e. after 90 min of incubation; Fig. 3C). Released GSH was quantified using Ellman's reagent (5,5′-dithiobis-(2-nitrobenzoic acid), DTNB) in the flow-through of ultrafiltered BAM1 samples. Soluble thiols were measured and found to be in a 1:1 molar ratio to BAM1 (Fig. 3D). The aim of this experiment was to investigate whether BAM1 glutathionylation could turn into an intramolecular disulfide bridge between 2 protein cysteines, with the release of free GSH. The results indicate that BAM1 is glutathionylated on a single cysteine and that this post-translational modification does not affect BAM1 activity per se. However, following a slow thiol–disulfide exchange reaction, GSH is released from glutathionylated BAM1 and a stable disulfide between 2 regulatory cysteines is formed, with consequent BAM1 inhibition.

### TRX f1 restores BAM1 activity after glutathionylation-mediated inhibition

We next attempted to recover BAM1 activity following the formation of regulatory disulfide promoted by glutathionylation. To this end, GSSG-pretreated BAM1 samples were first desalted to remove excess GSSG, then left to form the intramolecular disulfide and finally exposed to different reducing systems.

**Figure 4.**
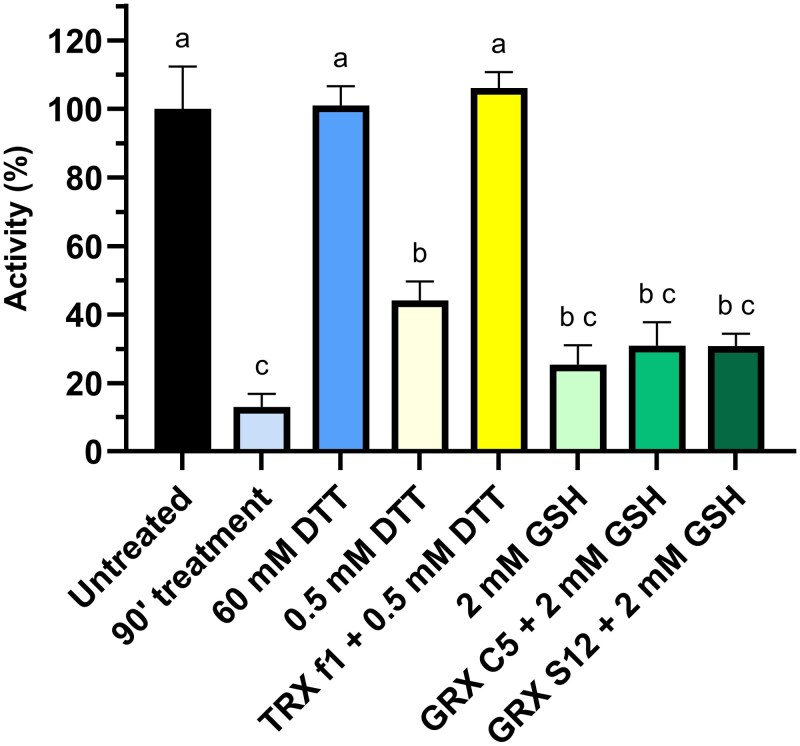
TRX f1 mediates rapid BAM1 reactivation. The reversibility of inactivation of BAM1 was assessed on inhibited BAM1 samples obtained after treatment with 1 mm GSSG for 1 h, desalted, and incubated for 90 min at 37 °C. Untreated sample followed the same procedure, except for the initial incubation that was with buffer. The recovery was tested incubating for 1 h with 1 *µ*M of TRX f1 and 0.5 mm DTT, GRX C5, or GRX S12 and 2 mm GSH. The experiment was carried out in triplicate; error bars show standard deviation. Data were analyzed with 1-way ANOVA and Tukey's test with *P* < 0.01, where distinct lowercase letters denote significant group differences.

As shown above, the formation of intramolecular disulfides after desalting decreased the activity by 85% compared to the control samples (Fig. 4, light blue bar). Subsequent incubation with 60 mm DTT for 1 h completely restored BAM1 activity (Fig. 4, blue bar). Alternatively, BAM1 activity was fully recovered by treatment with TRX f1 in the presence of 0.5 mm DTT as electron donor (Fig. 4, yellow bar). In contrast, the stromal Class I GRX C5 and GRX S12 (Couturier et al. 2011, 2009; Zaffagnini et al. 2012a, 2012b) allowed limited reactivation of BAM1 in the presence of 2 mm GSH (Fig. 4, green bar).

To assess if GRX could catalyze the reactivation of BAM1 upon longer incubations, the activity of BAM1 was followed for 16 h in the presence of GRXs and GSH (Supplementary Fig. S1). Under these conditions, the activity of BAM1 was completely recovered with either GSH alone or GRX plus GSH. Altogether, these data showed a strong preference for TRX f1 over GRX for BAM1 reactivation.

Considering that TRXs tend to be specific for protein disulfides (Cys–Cys, Michelet et al. 2013), while Class I GRXs are often involved in the reduction of GSH-protein mixed disulfides (Cys–GSH, Zaffagnini et al. 2012a, 2012b), these results strengthen the idea that the glutathionylation of BAM1 turns into an inhibitory intramolecular disulfide.

### Lower stromal GR activity affects BAM1 activity in vivo

*A. thaliana* encodes 2 isoforms of GSH reductase, both with dual localization, the cytoplasmic/peroxisomal GR1 and the plastidial/mitochondrial GR2 (Marty et al. 2019). To test if BAM1 may be regulated by GSH in vivo, we used *Arabidopsis* mutants with lower levels of plastidial/mitochondrial GR2 activity. Given the lethality of GR2 knock-out mutations, 2 lines with different residual levels of GR2 activity were used: *miao*, with only a few percent of wild-type GR2 activity (Yu et al. 2013) and *epc-2*, with less than 25% GR2 activity compared to wild type (Marty et al. 2019). As a negative control, *bam1* plants lacking BAM1 (Valerio et al. 2011) were also used.

**Figure 5.**
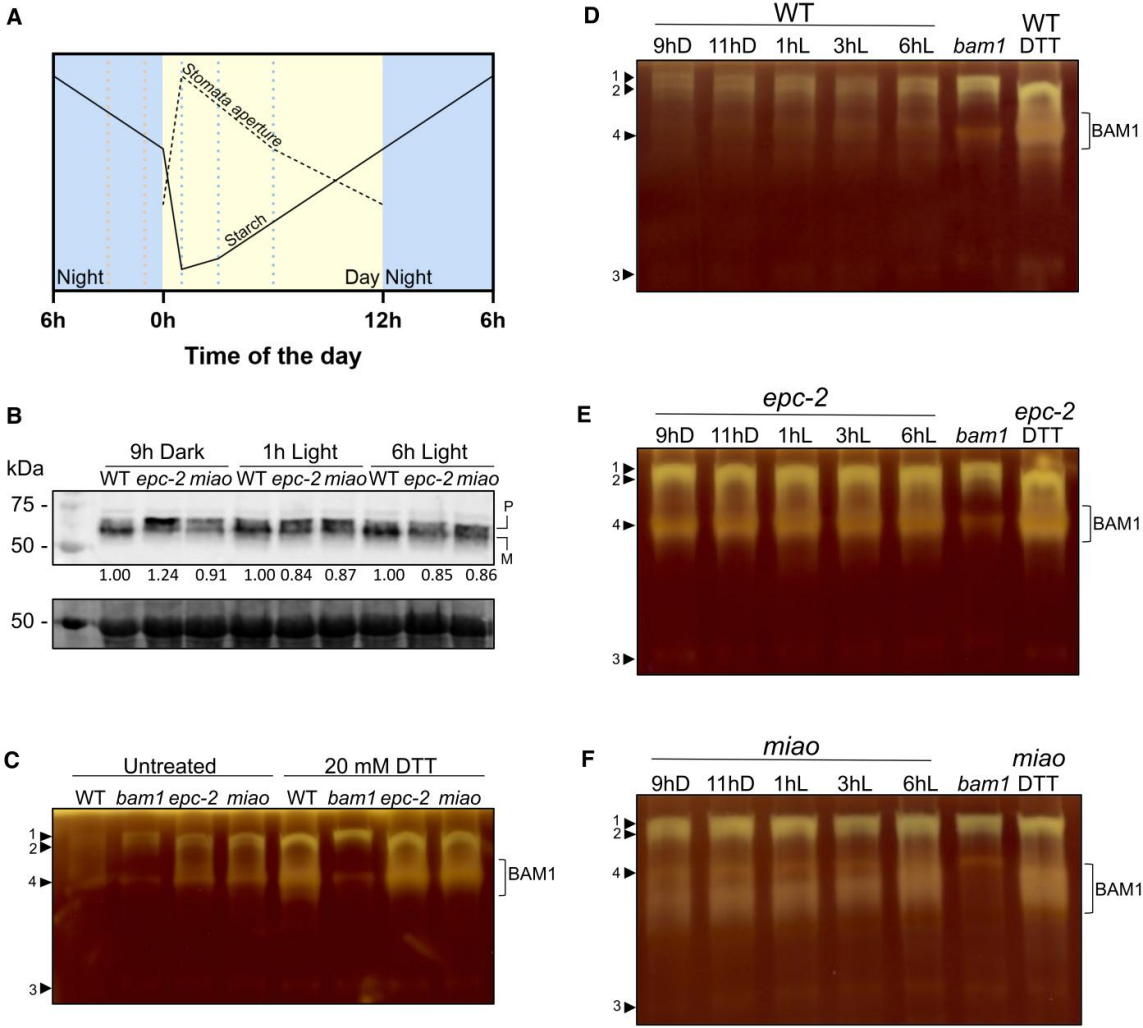
Alteration in GSH regeneration affects BAM1 activity. **A)** schematic representation of stomata aperture and starch levels in *Arabidopsis* chloroplasts of guard cells throughout the day. Adapted from Horrer et al. (2016). Sample collection times (9 and 11 h dark; 1, 3 and 9 h light) are marked by dotted lines. **B)** BAM1 protein levels visualized by Western blot using α-BAM1 antibodies. Protein extracts (40 *µ*g) from the indicated genotypes were prepared at different timepoints and loaded onto gels. BAM1 appears as 2 bands due to the presence of precursor (P) and mature BAM1 (M) in protein extracts (Feike et al. 2022). Ponceau staining of Rubisco large subunit is shown as loading control. The levels of total BAM1 protein (precursor + mature) were quantified with ImageJ, normalized on the wild-type value and shown as numbers at the bottom of the Western blot. **C)** In-gel amylolytic activity on native acrylamide gel with 0.1% amylopectin. Protein samples were collected at 1 h of light and incubated with extraction buffer (left side of the gel) or reduced DTT (right side of the gel) for 1 h before loading. Arrowheads indicate amylolytic activities (Bands 1, 2, 3, and 4) other than BAM1. **D)–F)** In-gel amylolytic activity on native acrylamide gel with 0.1% amylopectin from protein samples of the indicated genotype collected at the different time points. Part of the extract at 1 h of light was reduced with 20 mm DTT and 40 *µ*g of proteins were loaded onto gels to show the maximal activity. Arrowheads indicate amylolytic activities (Bands 1, 2, 3, and 4) other than BAM1.

Figure 5A illustrates a typical time course of starch concentration that reflects the ratio between the rate of starch synthesis and degradation. Whereas starch degradation predominates during the last 3 to 4 h of the night and accelerates during the first 3 h of daylight, starch synthesis dominates for the rest of the day until 6 h into the dark period (Horrer et al. 2016; Santelia and Lunn 2017).

To cover the period in which starch degradation is more active in guard cells, plants were collected at 18 days after germination (period in which the expression of BAM1 is confined to guard cells, Valerio et al. 2011) at 9 and 11 h of darkness, and 1, 3, and 6 h of light, respectively, Zeitgeber time (ZT) 21, ZT23, ZT3, and ZT6 in the 12 h light/12 h dark photoperiod.

First, the total amount of BAM1 was evaluated in soluble protein extracts from wild-type, *epc-2* and *miao* plants by Western blotting (Fig. 5B). The BAM1 level was generally higher in wild-type plants than in mutants (84% to 91% of wild type), as indicated by densitometric analysis of the bands performed with ImageJ (Schneider et al. 2012) and reported in Fig. 5B. The unique exception was *epc-2* at 9 h of darkness, with 124% of wild-type BAM1.

Protein extracts were then used to visualize hydrolytic activities on native gel containing amylopectin (Fig. 5, C to F). As controls, soluble protein extracts from *bam1* plants (negative control) and DTT-treated samples (fully activated samples) were loaded in all zymograms.

After DTT treatment, all genotypes exhibited an increased hydrolytic activity, with the most evident increase being shown by wild-type extracts (Fig. 5, C and D). Five major bands were identified in all zymograms. Bands 1 and 2, which, based on their position and appearance, should correspond to isoamylases (Delatte et al. 2005), increased their activity upon redox treatments in wild-type extract, while they appeared already active and less sensitive to DTT treatment in both *epc-2* and *miao* mutants. A third faint band at the bottom of the gel (Band 3) did not respond to reducing treatment, and a fourth band (Band 4), which occupied the same area of BAM1 activity, was visible in *bam1* line and partially masked in the other lines. Similar to Bands 1 and 2, the BAM1 signal increased strongly upon DTT treatment only in wild-type extract (Fig. 5D), while in the *epc-2* and *miao* mutants, it already appeared to be maximally activated regardless of the collection time (Fig. 5, E and F).

These findings indicate that BAM1 activity is higher under low GR2 activity, whereas it increases slightly from the dark to the light period only in wild-type plants.

### Redox balance of GSH affects starch levels in guard cells

It has been previously reported that BAM1 is mainly expressed in the guard cells of young plants (Valerio et al. 2011) and is responsible for approximately 80% of the amylolytic activity in guard cells (Horrer et al. 2016). Thus, to further test the effect of stromal GSH redox state on BAM1 activity in vivo, starch content in guard cells was assessed and used as a proxy measure for BAM1 activation state.

**Figure 6.**
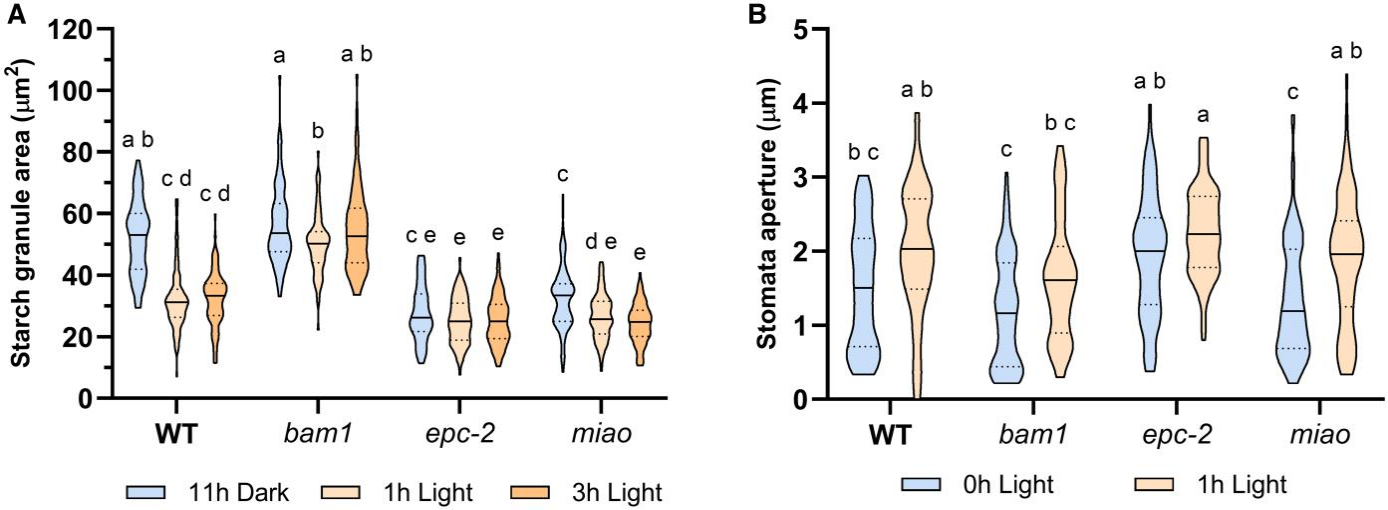
GR2 deficiency affects guard cell starch and stomata aperture. **A)** Starch area per guard cell in wild-type, *bam1*, *epc-2*, and *miao Arabidopsis* plants. **B)** Stomata aperture of wild-type, *bam1*, *epc-2*, and *miao* plants. Solid lines represent the median; dotted line represent the highest and the lowest quartiles. For starch granule area, 70 to 80 guard cells were analyzed at each time point, for stomata aperture, 60 to 90 pores were analyzed at each timepoint. Data were analyzed using 2-way ANOVA and Tukey's test with *P* < 0.01, where distinct lowercase letters denote significant group differences.

Epidermal peels from 18 days wild-type, *epc-2*, *miao* and *bam1* plants were collected at 11 h of dark and 1 and 3 h of light and stained for starch visualization. Pictures were recorded and the total area of starch granules in guard cells was quantified. In agreement with previous data (Valerio et al. 2011; Horrer et al. 2016), guard cells of *bam1* plants showed limited or no starch degradation during the first 3 h of the day, when guard cells of wild-type plants lose about 40% of their nighttime starch content (Fig. 6A). Consequently, *bam1* mutants contain more starch in guard cells than wild-type plants at the beginning of the day. In contrast, starch content in *epc-2* mutants was constantly lower than in wild-type plants (Fig. 6A) and did not change at the end of the night or during the day (Fig. 6A). Starch in *miao* mutants was intermediate between wild type and *epc-2*, as it was degraded in the dark-to-light transition but was invariably lower than in wild-type plants, similar to *epc-2* (Fig. 6A).

These complementary data confirm our results from in-gel activity assays: in the absence of BAM1 (Fig. 6A), starch granules are larger in guard cells. In wild-type guard cells, starch decreases after the onset of the day, whereas in *epc-2* and *miao* mutants, the constantly high BAM1 activity (Fig. 5, D to F) resulted in constitutively lower starch levels in guard cells (Fig. 6A).

### Stromal GSH homeostasis partially influences stomata aperture

Although starch degradation in guard cells starts in the last hours of the night, stomatal opening occurs in the first hour of light (Fig. 5A; Horrer et al. 2016). To complete the analysis of guard cells functioning under unbalanced GSH homeostasis, the opening of stomata was monitored at the end of the night (0 h light) and 1 h after the beginning of the day. All genotypes had a wider stomata aperture at 1 h light compared to darkness (0 h light; Fig. 6B). Comparing the genotypes, we observed that in the *epc-2* mutant stomata were more open than any other genotype both at the end of the night and at the beginning of the day. The stomata aperture of *miao* plants was comparable to wild type, while the aperture of *bam1* was the lowest among genotypes after 1 h of illumination.

## Discussion

Chloroplasts possess complex redox systems including diverse redox components (e.g. TRXs, GRXs, ROS, GSH, and ascorbate) (Souza et al. 2019). This study describes a redox regulatory mechanism involving BAM1 from *Arabidopsis*. The BAM1 from *Arabidopsis*, which is known to be activated by reduced TRXs in vitro (Sparla et al. 2006), can also interact with hydrogen peroxide and GSH. Because BAM1 is specifically expressed in guard cells of young *Arabidopsis* plants (Valerio et al. 2011), these regulatory properties of BAM1 should be considered when interpreting the altered stomatal physiology of mutants with reduced GR2 activity and consequent modification of GSH redox state.

### Dynamic redox regulation of BAM1 integrates inputs from GSH and TRX systems

We found that BAM1 is reversibly modified by hydrogen peroxide (Fig. 1A). This suggests an additional connection between BAM1 and photosynthesis, given the unavoidable generation of H_2_O_2_ by photosynthetic complexes in the light (Lee and Kim 2024). Even at the relatively high concentrations of H_2_O_2_ tested (0.5 mm, Fig. 1), we found that BAM1 can lose up to 70% of its activity and recover it completely upon reduction with DTT (Fig. 1), which clearly indicates that the formation of the inhibitory disulfide is fast enough to limit cysteine overoxidation to sulfinic/sulfonic acids, a common irreversible side effect of H_2_O_2_ regulation of enzyme activities (Zaffagnini et al. 2019; Cejudo et al. 2021; Yoshida and Hisabori 2023). We hypothesize that BAM1 may be adapted to conditions of oxidative stress such as mesophyll cells under osmotic/drought stress, in which *BAM1* expression was found to be strongly induced (Zanella et al. 2016). However, since the identity of BAM1 cysteines involved in redox PTM is still unknown, specific physiological studies involving cysteine mutants are not yet possible.

TRX f1 is one of the important redox transmitters involved in the synchronization of light reactions of photosynthesis and the activity of the Calvin–Benson–Bassham cycle (Michelet et al. 2013). In vitro experiments showed that BAM1 is preferentially reduced by TRX f1 among all major chloroplast TRX types (Sparla et al. 2006; Valerio et al. 2011). When TRX f1 reduction increases under light conditions (Zimmer et al. 2021; Hou et al. 2024), the inhibitory BAM1 disulfide decreases. Hydrogen peroxide, which is also produced in chloroplasts under light conditions, could directly contrast the activating effect of TRX f1 by promoting the formation of the disulfide. Alternatively, H_2_O_2_ may indirectly contribute to the same effect by acting as a terminal electron acceptor for the redox chain formed by atypical TRXs, TRXL2/ACHT and 2-cys peroxiredoxin, which may constitute a common route for dark-inactivation of light-activated enzymes (Zaffagnini et al. 2019; Yoshida and Hisabori 2023). In conclusion, the sensitivity to H_2_O_2_ appears to strengthen the coordination between BAM1 activity and photosynthesis, already sustained by TRX regulation.

Leaf tissue contains high levels of GSH (about 500 nmol/g fresh weight, low millimolar range concentration), which is kept in its reduced form (GSH) by the action of GSH reductases (GR1 in cytosol and peroxisomes and GR2 in plastids and mitochondria). In these compartments, in vivo GSH redox potential (*E*_GSH_) measurements using the genetically encoded biosensor roGFP2 revealed that the GSH:GSSG ratio ranges from 10,000 to 50,000:1, with GSSG concentrations between the micromolar and the nanomolar range (Schwarzländer et al. 2016; Müller-Schüssele et al. 2021).

**Figure 7.**
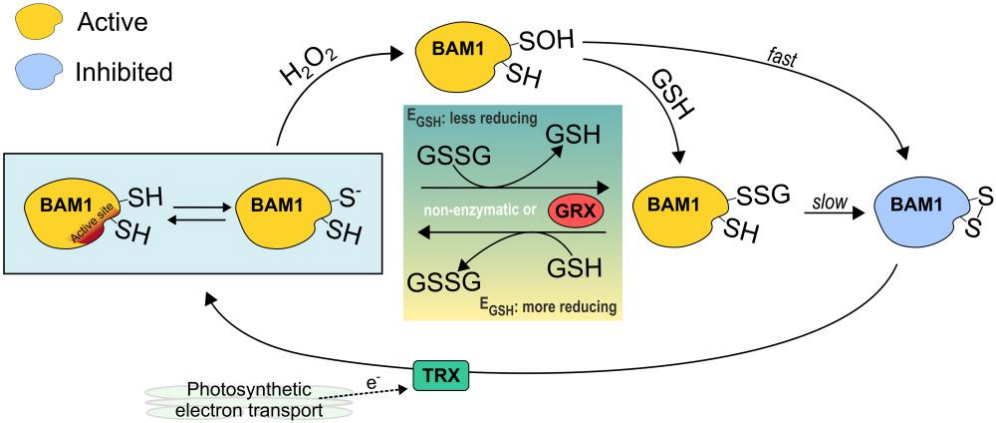
Model of BAM1 redox regulation. BAM1 reacts with H_2_O_2_, undergoing sulfenylation (–SOH). The sulfenylation is either removed by forming a disulfide bond with a cysteine from the active site, which inactivates BAM1, or by reacting with GSH, leading to glutathionylation (–SSG) and maintaining the enzyme's activity. Glutathionylation can be removed by the active site cysteine, leading to intramolecular disulfide bond and inhibition of activity. Glutathionylation can occur also by direct reaction with oxidized GSH (GSSG). GRXs keep target cysteine redox states and GSH redox state near thermodynamic equilibrium. Depending on the GSH redox potential (*E*_GSH_), GRXs can catalyze glutathionylation or deglutathionylation. The inhibitory intramolecular disulfide bond can be reduced by chloroplast TRX using the reducing power provided by photosynthesis.

The high concentration of GSH in plant cells makes it possible that protein cysteines initially attacked by H_2_O_2_ may react with GSH, resulting in protein glutathionylation (Fig. 7). Indeed, we observed that simultaneous incubation with GSH and H_2_O_2_ led to BAM1 glutathionylation, and the same modification could be obtained by incubating the enzyme with GSSG (Fig. 2, A and B). However, glutathionylation itself did not affect BAM1 activity, not directly at least (Fig. 2C). These data agree with the work of Storm and colleagues (2018), which showed that *S*-nitrosoglutathione treatment led to glutathionylation of BAM1 without influencing the catalysis. In vitro after the removal of the glutathionylating reagent and prolonged incubation, glutathionylated BAM1 became inactive due to the formation of a disulfide (Figs. 3C and 4). Several evidence support the transient nature of BAM1 glutathionylation: (i) the disappearance of the GSH signal in Western blots analysis after the removal of the glutathionylating reagent (Fig. 3, A and B) and (ii) the concomitant release in solution of 1 mole of GSH per mole of BAM1 (Fig. 3D); (iii) the inhibition of BAM1 activity observed only after the removal of GSSG or H_2_O_2_ and GSH (Fig. 3C) and (iv) the fast recovery of BAM1 activity by TRX f1 but not by GRX S12 or C5 (Fig. 4). Given that transient glutathionylation does not inactivate BAM1, the glutathionylation site should be outside the active site in a position that does not interfere with the catalysis. Conversely, the resolving cysteine, which attacks the mixed disulfide of the glutathionylated cysteine, is likely to be inside the catalytic pocket (Fig. 7), as its involvement in a disulfide bond leads to the inhibition of the activity. Overall, the mechanism identified here constitutes an alternative way to control BAM1 catalysis, at the intersection between H_2_O_2_/GSH and TRXs, highlighting the complexity and the complementarity of redox systems in chloroplasts.

### Effects of altered GSH redox state on BAM1 activity in vivo

With the aim of investigating whether the GSH-dependent modulation of BAM1 activity could exist in vivo, the behavior of BAM1 was analyzed in leaf extracts of plants with altered plastid GR2 activity (*epc-2* and *miao*).

By comparing the in-gel activity of leaf extracts of wild-type plants and GR2 mutants, it was clear that *epc-2* and *miao* had higher BAM1 activity at all time points analyzed (Fig. 5, D to F). Considering that the midpoint redox potential of the regulatory disulfide of BAM1 is—360 mV at pH 8 (Sparla et al. 2006) and that the GSH redox potential in chloroplasts (*E*_GSH_) is between—310 and 365 mV (Schwarzländer et al. 2008; Rosenwasser et al. 2010; Müller-Schüssele et al. 2020; Haber et al. 2021), even slight changes in GSSG levels, as expected in GR2 mutants, should influence the propensity of GSH to modify BAM1. A decrease in the [GSH]/[GSSG] ratio might also slow down the rate of deglutathionylation by GRXs (Zaffagnini et al. 2012a, 2012b; Zaffagnini et al. 2019), thus keeping BAM1 in the glutathionylated state for a longer time. Increased steady-state levels of glutathionylated BAM1 would result in a higher pool of enzyme molecules transitorily blocked in an active state, though ready to be inhibited by homodisulfide formation. In the case of *epc-2* and *miao*, it is possible to hypothesize that *E*_GSH_ is constantly more positive that in wild-type plants, so that BAM1 would be more active, as suggested by our in-gel activity assays (Fig. 5, C to F) because of glutathionylation and thus, protection from oxidants.

### In vivo consequences of altered GSH homeostasis on guard cells starch and stomata aperture

In young and unstressed plants, *BAM1* expression is confined to guard cells (Valerio et al. 2011; Zanella et al. 2016) and starch content in these specialized cells has been used as a proxy to analyze BAM1 activation. Starch content was quantified after 11 h of darkness and after 1 and 3 h of illumination. As previously observed, starch degradation in *Arabidopsis* guard cells proceeds during the last hours of the night (i.e. it should be active at 11 h darkness) and the first hours of the day, then it tends to decrease after 3 h of illumination when starch synthesis prevails (Horrer et al. 2016; Santelia and Lunn 2017). As expected, plants lacking BAM1 were unable to degrade starch in guard cells and to completely open the stomatal pore (Fig. 6, A and B). On the other hand, guard cells of both GR2 mutants were found to contain much less starch at all time points (Fig. 6A), in agreement with the activated state of BAM1 observed in-gel (Fig. 5, E and F). The high activity of BAM1 at the end of the night in these plants allows us to hypothesize that GSH could enable the night activation of BAM1, although studies are needed to characterize the physiological nighttime regulation of BAM1.

Different from *bam1* mutants, which limit the opening of stomata in the light possibly because of their low starch degradation, *epc-2* mutants keep stomata in a relatively open state even in the dark (Fig. 6B), in agreement with their low levels of starch in guard cells (Fig. 6A). Therefore, by comparing *bam1* and *epc-2* mutants, a coherent inverse correlation was observed between starch content (related to BAM1 activity) and stomata aperture.

A similar inverse correlation was not apparent in *miao* mutants, which contain less starch than wild type in guard cells, but more than *epc-2* at 11 h of dark (Fig. 6A), and opens stomata like wild-type plants (Fig. 6B). Different reasons can be hypothesized for this discrepancy. Many steps are involved in the molecular mechanism that induces changes in turgor pressure in guard cells (ROS production, blue light perception, H^+^ and K^+^ pumping, and malate production; Lemonnier and Lawson 2024). The GSH redox state has been observed to exert an influence on these processes, acting as a negative regulator of abscisic acid signal components (Okuma et al. 2011; Akter et al. 2012). Moreover, it is noteworthy that severe defects have been found in the root apical meristem of *miao* plants, leading to a strong inhibition of root growth (Yu et al. 2013). Short roots could possibly impair water balance and influence guard cell function. In addition, the effect of low GR2 activity in mithochondria should be considered.

In the context of *epc-2* plants, it is important to note that these plants are completely devoid of GR2 in mitochondria, even though GSH and TRX systems can compensate each other to a certain extent in this organelle (Marty et al. 2019). The energy from mitochondria is needed for stomata aperture, especially for the ion pumps involved, then the absence of GR2 in mitochondria may potentially give rise to hitherto unknown effects on stomatal aperture. Of course, a combination of all the factors mentioned should be taken into account.

Although further research is needed to detail how physiological and environmental signals modulate GSH-mediated regulation of BAM1, here we show that BAM1 can function as redox hub integrating inputs from TRX and GSH systems.

## Conclusions

The present study demonstrates that the activity of BAM 1 of *A. thaliana* is additionally influenced by the GSH redox system. Although BAM1 can undergo glutathionylation, this PTM itself does not affect enzyme activity directly but rather slows down the inactivation rate of BAM1 in response to H_2_O_2_. Class I GRXs remove the glutathionylation, restoring the reduced cysteinyl thiol. However, the glutathionylation event can slowly proceed through the formation of the inhibitory regulatory disulfide and the concomitant release of GSH.

After inhibitory disulfide formation, BAM1 reactivation can be performed by TRX f1. The analysis of GR2-defective lines showed that stromal GSH redox state affects BAM1 activity in vivo, resulting in a more active enzyme. In our current view, the higher activation state of BAM1 in GR2 mutants compared to wild-type plants would depend on a slower rate of the formation of the regulatory intramolecular disulfide due to higher glutathionylation, or lower deglutathionylation, rates. Combining these results show an example of glutathionylation as a purely protective mechanism, interfering with the formation of an inhibitory disulfide bond and keeping the enzyme more active.

Here we show that stromal GSH redox state influences starch metabolism, at least in part, through BAM1 activation state. In particular, these initial investigations into starch metabolism under altered stromal GSH homeostasis have highlighted the necessity to further clarify the role of GSH in this process and its relationship with stomatal function. Given the importance of stomata for maintaining plant water balance and growth, and the role of starch in stomata physiology, these results pave the way for the discovery of mechanisms of redox-mediated regulation of stomata opening.

## Materials and methods

### Expression and purification of recombinant AtBAM1

Wild-type BAM1 was expressed and purified, as described in Sparla et al. (2006). Briefly, *Escherichia coli* BL21 (DE3) cells, harboring the expression vector pET28 (Novagen-Merck) containing the coding sequence for the mature form of BAM1 in frame with an His-tag at the N-terminus, were grown at 37 °C before and after the induction of expression with 0.4 mm isopropyl β-D-1-thiogalactopyranoside. Cells were collected by centrifugation and stored at −80 °C before purification procedure. Purification was conducted by loading the soluble fraction of sonicated cells onto Chelating Sepharose Fast Flow (Cytiva). Binding, washing, and elution steps were performed following the manufacturer's instruction and applying buffer with increasing imidazole concentration (i.e. 5, 60, and 500 mm).

After purification, BAM1 was desalted in 30 mm Tris–HCl pH 7.9, 1 mm EDTA; purity was assessed by 12.5% SDS–PAGE gel and the His-Tag was removed by Thrombin Protease (Cytiva).

Pure recombinant BAM1 was quantified by absorbance at 280 nm (Nanodrop N-1000; Thermo Fisher Scientific) using a molar extinction coefficient of 99,030 m^−1^ cm^−1^ and molecular mass of 59,721 Da calculated on the primary sequence through ProtParam (Wilkins et al. 1999). *A. thaliana* TRX f1 was generously gifted by Emmanuelle Issakidis-Bourguet (CNRS-University of Paris Saclay, France). *A. thaliana* GRX C5 and *Populus tremula x tremuloides* GRX S12 were kindly provided by Nicolas Rouhier (University of Nancy, France).

### Activity measurements and redox treatments

Catalytic activity was assayed incubating 60 *µ*l of 1 *µ*M BAM1 with an equal volume of the artificial substrate *p*-nitrophenyl maltotrioside (PNPβ-G3; Betamyl3, Megazyme, Ireland) at 40 °C for 10 min, then the reaction was stopped with 900 *µ*l of stop solution (Tris 1%, pH 8.5) following the manufacturer's instructions. The absorption of the p-nitrophenyl group released by the reaction was measured at 400 nm in a standard spectrophotometer (Cary 60, Agilent).

For oxidative treatments, BAM1 was pre-reduced with 20 mm DTT at 37 °C for 1 h, and then desalted in 100 mm Tricine pH 7.9 using NAP5 columns (Cytiva). Pre-reduced BAM1 was incubated in 100 mm Tricine–NaOH pH 7.9 at 25 °C in the absence (control) or presence of 0.5 mm H_2_O_2_, 0.5 mm H_2_O_2_ plus 2.5 mm reduced GSH or 1 mm oxidized GSH (GSSG) for 1 h at 25 °C. Recovery from hydrogen peroxide was assessed by incubating with 60 mm DTT for 30 min at 37 °C. Inactivation kinetics were performed incubating BAM1 at 25 °C with or without (control) 0.5 mm H_2_O_2_ and 0.5 mm H_2_O_2_ plus 2.5 mm GSH; the activity was assayed at the indicated time points activity.

To demonstrate that the same cysteine can be glutathionylated by H_2_O_2_ and GSH or GSSG, 30 *µ*M BAM1 was incubated in presence of 1 mm GSSG for 1 h. Then, the incubation was diluted 2 times and incubated with 20 mm IAM and 20 mm NEM for 30 min to block the remaining free cysteines. The solution was further diluted 5 times and incubated with 40 mm reduced DTT for 30 min. At the end of the reduction, the sample was desalted through a NAP5 column (Cytiva) and incubated with 0.5 mm H_2_O_2_ and 2. 5 mm GSH. At every step, the equivalent of 1 *µ*g of BAM1 protein was collected for Western blot analysis with α-GSH antibodies (see *Western blot analysis* section).

For the deglutathionylation assay, BAM1 was treated with 1 mm GSSG, and after 1 h it was desalted through a NAP5 column in 100 mm Tricine pH 7.9. Then, BAM1 was incubated for 30 min with 0.5 mm DTT; 2 mm GSH; 2 mm GSH; and 1 *µ*M *A. thaliana* GRX C5 or 1 *µ*M *P. tremula x tremuloides* GRX S12. The incubation was stopped by the addition of nonreducing loading buffer for SDS–PAGE and then analyzed on Western blot with α-GSH antibodies (see *Western blot analysis* section).

To assay inhibition caused by loss of GSH, pre-reduced samples of BAM1 treated with 1 mm GSSG or 0.5 mm H_2_O_2_ plus 2.5 mm GSH at 25 °C were desalted after 1 h through NAP5 column. Desalted samples were incubated at 37 °C and the activity was assayed at the indicated time points and normalized on the activity of BAM1 at 0 min.

To assess the reactivation of BAM1, the enzyme was treated with 1 mm GSSG for 1 h, then desalted through a NAP5 column in 100 mm Tricine pH 7.9, and incubated at 37 °C for 90 min to induce the inhibition. Then, inhibited BAM1 was incubated at 25 °C for 1 h in the presence of 60 mm or 0.5 mm DTT; 0.5 mm DTT and 1 *µ*M *A. thaliana* TRX f1; GSH 2 mm; GSH 2 mm and 1 *µ*M *A. thaliana* GRX C5 or 1 *µ*M *P. tremula x tremuloides* GRX S12.

### Quantification of released GSH in BAM1 medium

The amount of GSH released from glutathionylated BAM1 was determined, as described in Gurrieri et al. (2019). In brief, BAM1 was treated with 1 mm GSSG for 1 h at room temperature and then desalted in 100 mm Tricine pH 7.9 through NAP5 column. After 90 min of incubation at 37 °C, protein samples were filtered using Amicon Ultra (Millipore, 10 kDa cutoff) and soluble thiols were quantified in the flow-through by incubation for 15 min with 0.1 mm DTNB at room temperature. The number of released thiols was calculated from the absorbance of 2-nitro-5-thiobenzoate(thiolate)dianion (ε_412nm_ 14,150 m^−1^ cm^−1^) and expressed as a molar ratio to the concentration of pre-filtered BAM1.

### Plant material

To test the regulatory role of GSH on BAM1 in vivo, *Arabidopsis* plants with reduced GR 2 activity were analyzed. GR2 has a double localization in chloroplast and mitochondria (Creissen et al. 1995; Chew et al. 2003), and knockout mutants are embryo lethal. Therefore, we took advantage of 2 *Arabidopsis* lines with reduced levels of GR2 activity, i.e. *epc-2* and *miao*. The *epc-2* mutant has been generated by Marty and colleagues (2019), this line is a *gr2* knockout plant complemented by a very low GR2 expression in plastids, while the *miao* line bears a missense mutation that severely impairs GR2 activity (Yu et al. 2013).

Wild type (Columbia-0), *bam1*, *miao*, and *epc-2* plants were grown on soil in a growth chamber at a constant temperature of 22 °C and 12 h/12 h light/dark cycle. The light intensity was of 100 to 120 *μ*mol photons m^−2^ s^−1^. Seeds were stratified at 4 °C for 3 days before being placed in the growth chamber. *bam1* seeds were selected in Valerio et al. (2011). Plants were collected at the 6-leaf stage, corresponding to 18-day old plants on average; at this stage, the expression of BAM1 is confined to guard cells (Valerio et al. 2011).

### In-gel amylolytic activity

Soluble proteins were extracted in 100 mm MOPS pH 7.2, 1 mm EDTA, 10% glycerol, 5 mm β-mercaptoethanol, and 1 mm phenylmethylsulfonyl fluoride using a pestle and a 1:2 fresh weight:extraction buffer ratio. Insoluble material was pelleted at 13,000 × *g* for 15 min at 4 °C. Soluble protein concentration was quantified by Bradford assay (Bradford 1976).

A part of protein extracts was treated with 20 mm DTT and incubated for 1 h at room temperature. Then 40 *µ*g of soluble proteins from untreated and treated extracts were mixed with native loading buffer (60 mm Tris pH 6.8; 10% glycerol; and 0.0025% bromophenol blue) and loaded onto a 7.5% acrylamide gel containing 0.1% (w/v) amylopectin from potato starch (Merck). Gels were run at 25 mA for 2 h in ice. After separation, gels were washed in 100 mm Tris–HCl pH 7; 1 mm MgCl_2_; and 1 mm CaCl_2_ for 15 min and subsequently incubated for 4 h at 37 °C in the same buffer. After the incubation, gels were washed with bidistilled water and stained with Lugol's solution (0.33% I_2_ and 0.66% KI).

### Western blot analysis

To test the glutathionylation of BAM1 after 0.5 mm H_2_O_2_ plus 2.5 mm GSH or 1 mm GSSG treatment, 2 *µ*g of protein sample were taken from incubations and were separated on nonreducing SDS–PAGE at 12.5% acrylamide. Proteins were transferred from gel to 0.2 *µ*m nitrocellulose membrane using TransBlot Turbo system (Bio-Rad). Glutathionylation was tested using 1:1,000 α-GSH monoclonal antibodies (101-A, Virogen) and 1:2,500 peroxidase-conjugated α-mouse, diluted in 20 mm Tris pH 7.4, 0.9% NaCl, and 0.1% Tween-20 (TBST), 3% nonfat skim milk. After the transfer, the membrane was blocked with 3% nonfat skim milk for 1 h in TBST, then incubated overnight with α-GSH antibodies at 4 °C. The following day, the membrane was washed with 3% nonfat skim milk for 1 h in TBST and incubated with peroxidase-conjugated α-mouse antibodies for 3 h, then washed with 3% nonfat skim milk in TBST. The chemiluminescence signal was detected using the Amersham ECL Western Blotting Detection Reagent (Cytiva) on ImageQuant LAS 500 (Cytiva).

For the detection of BAM1 protein levels, *Arabidopsis* extracts were prepared as mentioned earlier, then 40 *µ*g of total soluble protein were loaded on SDS–PAGE. The α-BAM1 antibody (Agrisera) was diluted at 1:7,500 and secondary antibody α-rabbit at 1:10,000.

### Guard cell starch quantification

Epidermal peels were collected from leaves at 11 h of darkness and 1 and 3 h of light, incubated in fixing solution (50% methanol and 10% acetic acid) overnight at 4 °C. Samples were rinsed in bidistilled water 2 times, and the starch was stained with Lugol’s solution prior to visualization using a light microscope (Nikon, SMZ1000). The starch granule area was calculated using ImageJ software (Schneider et al. 2012).

### Stomatal aperture

For stomatal aperture measurement, a fully developed leaf was cut at the indicated time points from each plant and fixed on double-sided tape attached to the glass slide. The abaxial epidermis of each sample was obtained by removing the rest of the leaf with a scalpel. The epidermis on the slide was washed with 3 ml of 10 mm MES pH 6.15 to remove cell debris and pictures of stomata were immediately taken at 40× magnification (Nikon, SMZ1000). The obtained pictures were analyzed using ImageJ (Schneider et al. 2012). A total of 60 to 80 stomata were measured from 3 biological replicates at each time point.

### Accession numbers

Sequence data from this article can be found in the GenBank/EMBL data libraries under accession numbers NM113297.3/Q9LIR6.

## Supplementary Material

kiaf344_Supplementary_Data

## Data Availability

The data underlying this article are available in AMS Acta repository of the University of Bologna, at https://amsacta.unibo.it/id/eprint/8477.

## Supplementary data

The following materials are available in the online version of this article.

**Supplementary Figure S1.** GSH- and GRX-dependent recovery of BAM1 activity requires prolonged incubation.

## Dive Curated Terms

The following phenotypic, genotypic, and functional terms are of significance to the work described in this paper:

NTRC Gramene: AT2G41680
NTRC Araport: AT2G41680

starch CHEBI: CHEBI:28017

BAM1 Gramene: AT3G23920
BAM1 Araport: AT3G23920

AMY3 Gramene: AT1G69830
AMY3 Araport: AT1G69830

GR2 Gramene: AT3G54660
GR2 Araport: AT3G54660

## References

[kiaf344-B1] Akter N, Sobahan MA, Uraji M, Ye W, Hossain MA, Mori IC, Nakamura Y, Murata Y. Effects of depletion of glutathione on abscisic acid and methyl jasmonate-induced stomatal closure in *Arabidopsis thaliana*. Biosci Biotechnol Biochem. 2012:76(11):2032–2037. 10.1271/bbb.120384 23132563

[kiaf344-B2] Ballicora MA, Frueauf JB, Fu Y, Schü Rmann P, Preiss J. Activation of the potato tuber ADP-glucose pyrophosphorylase by thioredoxin. J Biol Chem. 2000:275(2):1315–1320. 10.1074/jbc.275.2.1315 10625679

[kiaf344-B3] Bohle F, Rossi J, Tamanna SS, Jansohn H, Schlosser M, Reinhardt F, Brox A, Bethmann S, Kopriva S, Trentmann O, et al Chloroplasts lacking class I glutaredoxins are functional but show a delayed recovery of protein cysteinyl redox state after oxidative challenge. Redox Biol. 2024:69:103015. 10.1016/j.redox.2023.103015 38183796 PMC10808970

[kiaf344-B4] Bradford MM . A rapid and sensitive method for the quantitation of microgram quantities of protein utilizing the principle of protein-dye binding. Anal Biochem. 1976:72(1–2): 248–254. 10.1016/0003-2697(76)90527-3 942051

[kiaf344-B5] Cejudo FJ, Sandalio LM, Van Breusegem F. Understanding plant responses to stress conditions: redox-based strategies. J Exp Bot. 2021:72(16): 5785–5788. 10.1093/jxb/erab324 34378048 PMC8355751

[kiaf344-B6] Chew O, Whelan J, Millar AH. Molecular definition of the ascorbate-glutathione cycle in Arabidopsis mitochondria reveals dual targeting of antioxidant defenses in plants. J Biol Chem. 2003:278(47):46869–46877. 10.1074/jbc.M307525200 12954611

[kiaf344-B7] Corpas FJ, González-Gordo S, Rodríguez-Ruiz M, Muñoz-Vargas MA, Palma JM. Thiol-based oxidative posttranslational modifications (OxiPTMs) of plant proteins. Plant Cell Physiol. 2022:63(7): 889–900. 10.1093/pcp/pcac036 35323963 PMC9282725

[kiaf344-B8] Couturier J, Koh CS, Zaffagnini M, Winger AM, Gualberto JM, Corbier C, Decottignies P, Jacquot JP, Lemaire SD, Didierjean C, et al Structure-function relationship of the chloroplastic glutaredoxin S12 with an atypical WCSYS active site. J Biol Chem. 2009:284(14):9299–9310. 10.1074/jbc.M807998200 19158074 PMC2666582

[kiaf344-B9] Couturier J, Ströher E, Albetel AN, Roret T, Muthuramalingam M, Tarrago L, Seidel T, Tsan P, Jacquot JP, Johnson MK, et al Arabidopsis chloroplastic glutaredoxin C5 as a model to explore molecular determinants for iron-sulfur cluster binding into glutaredoxins. J Biol Chem. 2011:286(31):27515–27527. 10.1074/jbc.M111.228726 21632542 PMC3149344

[kiaf344-B10] Creissen G, Reynolds H, Xue Y, Mullineaux P. Simultaneous targeting of pea glutathione reductase and of a bacterial fusion protein to chloroplasts and mitochondria in transgenic tobacco. Plant J. 1995:8(2):167–175. 10.1046/j.1365-313X.1995.08020167.x 7670502

[kiaf344-B901] Daloso DM, Medeiros DB, Dos Anjos L, Yoshida T, Araújo WL, Fernie AR . Metabolism within the specialized guard cells of plants. New Phytol. 2017:216(4):1018–1033. 10.1111/nph.14823. Epub 2017 Oct 6. PMID: 28984366. 28984366

[kiaf344-B11] Delatte T, Trevisan M, Parker ML, Zeeman SC. Arabidopsis mutants Atisa1 and Atisa2 have identical phenotypes and lack the same multimeric isoamylase, which influences the branch point distribution of amylopectin during starch synthesis. Plant J. 2005:41(6):815–830. 10.1111/j.1365-313X.2005.02348.x 15743447

[kiaf344-B12] Deponte M . The incomplete glutathione puzzle: just guessing at numbers and figures? Antioxid Redox Signal. 2017:27(15):1130–1161. 10.1089/ars.2017.7123 28540740 PMC5661824

[kiaf344-B13] Dittrich P, Raschke K. Malate metabolism in isolated epidermis of *Commelina communis* L. in relation to stomatal functioning. Planta. 1977:134(1):77–81. 10.1007/BF00390098 24419583

[kiaf344-B902] Feike D, Pike M, Gurrieri L, Graf A, Smith AM . A dominant mutation in β–AMYLASE1 disrupts nighttime control of starch degradation in Arabidopsis leaves. Plant Physiol. 2022:188(4):1979–1992. 10.1093/plphys/kiab603 34958379 PMC8968401

[kiaf344-B14] Fernandez O, Ishihara H, George GM, Mengin V, Flis A, Sumner D, Arrivault S, Feil R, Lunn JE, Zeeman SC, et al Leaf starch turnover occurs in long days and in falling light at the end of the day. Plant Physiol. 2017:174(4):2199–2212. 10.1104/pp.17.00601 28663333 PMC5543966

[kiaf344-B15] Flütsch S, Horrer D, Santelia D. Starch biosynthesis in guard cells has features of both autotrophic and heterotrophic tissues. Plant Physiol. 2022:189(2):541–556. 10.1093/plphys/kiac087 35238373 PMC9157084

[kiaf344-B16] Flütsch S, Wang Y, Takemiya A, Vialet-Chabrand SRM, Klejchová M, Nigro A, Hills A, Lawson T, Blatt MR, Santelia D. Guard cell starch degradation yields glucose for rapid stomatal opening in Arabidopsis. Plant Cell. 2020:32(7):2325–2344. 10.1105/tpc.18.00802 32354788 PMC7346545

[kiaf344-B17] Foyer CH, Noctor G. Ascorbate and glutathione: the heart of the redox hub. Plant Physiol. 2011:155(1): 2–18. 10.1104/pp.110.167569 21205630 PMC3075780

[kiaf344-B18] Gurrieri L, Distefano L, Pirone C, Horrer D, Seung D, Zaffagnini M, Rouhier N, Trost P, Santelia D, Sparla F. The thioredoxin-regulated α-Amylase 3 of *Arabidopsis thaliana* is a target of S-glutathionylation. Front Plant Sci. 2019:10:993. 10.3389/fpls.2019.00993 31417599 PMC6685290

[kiaf344-B19] Gurrieri L, Fermani S, Zaffagnini M, Sparla F, Trost P. Calvin–Benson cycle regulation is getting complex. Trends Plant Sci. 2021:26(9):898–912. 10.1016/j.tplants.2021.03.008 33893047

[kiaf344-B20] Gurrieri L, Sparla F, Zaffagnini M, Trost P. Dark complexes of the Calvin-Benson cycle in a physiological perspective. Semin Cell Dev Biol. 2024:155:48–58. 10.1016/j.semcdb.2023.03.002 36889996

[kiaf344-B21] Haber Z, Lampl N, Meyer AJ, Zelinger E, Hipsch M, Rosenwasser S. Resolving diurnal dynamics of the chloroplastic glutathione redox state in Arabidopsis reveals its photosynthetically derived oxidation. Plant Cell. 2021:33(5):1828–1844. 10.1093/plcell/koab068 33624811 PMC8254480

[kiaf344-B22] Horrer D, Flütsch S, Pazmino D, Matthews JSA, Thalmann M, Nigro A, Leonhardt N, Lawson T, Santelia D. Blue light induces a distinct starch degradation pathway in guard cells for stomatal opening. Curr Biol. 2016:26(3):362–370. 10.1016/j.cub.2015.12.036 26774787

[kiaf344-B23] Hou LY, Sommer F, Poeker L, Dziubek D, Schroda M, Geigenberger P. The impact of light and thioredoxins on the plant thiol-disulfide proteome. Plant Physiol. 2024:195(2):1536–1560. 10.1093/plphys/kiad669 38214043 PMC11142374

[kiaf344-B24] Ishihara H, Alseekh S, Feil R, Perera P, George GM, Niedzwiecki P, Arrivault S, Zeeman SC, Fernie AR, Lunn JE, et al Rising rates of starch degradation during daytime and trehalose 6-phosphate optimize carbon availability. Plant Physiol. 2022:189(4):1976–2000. 10.1093/plphys/kiac162 35486376 PMC9342969

[kiaf344-B25] Lee KP, Kim C. Photosynthetic ROS and retrograde signaling pathways. New Phytol. 2024:244(4):1183–1198. 10.1111/nph.20134 39286853

[kiaf344-B26] Lemonnier P, Lawson T. Calvin cycle and guard cell metabolism impact stomatal function. Semin Cell Dev Biol. 2024:155:59–70. 10.1016/j.semcdb.2023.03.001 36894379

[kiaf344-B27] Lloyd JR, Kossmann J, Ritte G. Leaf starch degradation comes out of the shadows. Trends Plant Sci. 2005:10(3):130–137. 10.1016/j.tplants.2005.01.001 15749471

[kiaf344-B28] Marty L, Bausewein D, Müller C, Bangash SAK, Moseler A, Schwarzländer M, Müller-Schüssele SJ, Zechmann B, Riondet C, Balk J, et al Arabidopsis glutathione reductase 2 is indispensable in plastids, while mitochondrial glutathione is safeguarded by additional reduction and transport systems. New Phytol. 2019:224(4):1569–1584. 10.1111/nph.16086 31372999

[kiaf344-B29] Michelet L, Zaffagnini M, Morisse S, Sparla F, Pérez-Pérez ME, Francia F, Danon A, Marchand CH, Fermani S, Trost P, et al Redox regulation of the Calvin-Benson cycle: something old, something new. Front Plant Sci. 2013:4:470. 10.3389/fpls.2013.00470 24324475 PMC3838966

[kiaf344-B30] Mikkelsen R, Mutenda KE, Mant A, Schü Rmann P, Blennow A. Glucan, water dikinase (GWD): a plastidic enzyme with redox-regulated and coordinated catalytic activity and binding affinity. Proc Natl Acad Sci U S A. 2005:102(5):1785–1790. 10.1073/pnas.0406674102 15665090 PMC547843

[kiaf344-B31] Müller-Schüssele SJ, Schwarzländer M, Meyer AJ. Live monitoring of plant redox and energy physiology with genetically encoded biosensors. Plant Physiol. 2021:186(1): 93–109. 10.1093/plphys/kiab019 34623445 PMC8154060

[kiaf344-B32] Müller-Schüssele SJ, Wang R, Gütle DD, Romer J, Rodriguez-Franco M, Scholz M, Buchert F, Lüth VM, Kopriva S, Dörmann P, et al Chloroplasts require glutathione reductase to balance reactive oxygen species and maintain efficient photosynthesis. Plant J. 2020:103(3):1140–1154. 10.1111/tpj.14791 32365245

[kiaf344-B33] Noctor G, Reichheld JP, Foyer CH. ROS-related redox regulation and signaling in plants. Semin Cell Dev Biol. 2018:80:3–12. 10.1016/j.semcdb.2017.07.013 28733165

[kiaf344-B34] Okuma E, Jahan MS, Munemasa S, Hossain MA, Muroyama D, Islam MM, Ogawa K, Watanabe-Sugimoto M, Nakamura Y, Shimoishi Y, et al Negative regulation of abscisic acid-induced stomatal closure by glutathione in Arabidopsis. J Plant Physiol. 2011:168(17):2048–2055. 10.1016/j.jplph.2011.06.002 21764168

[kiaf344-B35] Paulsen CE, Carroll KS. Cysteine-mediated redox signaling: chemistry, biology, and tools for discovery. Chem Rev. 2013:113(7): 4633–4679. 10.1021/cr300163e 23514336 PMC4303468

[kiaf344-B36] Rodrigues O, Shan L. Stomata in a state of emergency: H_2_O_2_ is the target locked. Trends Plant Sci. 2022:27(3):274–286. 10.1016/j.tplants.2021.10.002 34756808

[kiaf344-B37] Rosenwasser S, Rot I, Meyer AJ, Feldman L, Jiang K, Friedman H. A fluorometer-based method for monitoring oxidation of redox-sensitive GFP (roGFP) during development and extended dark stress. Physiol Plant. 2010:138(4):493–502. 10.1111/j.1399-3054.2009.01334.x 20051029

[kiaf344-B38] Santelia D, Lawson T. Rethinking guard cell metabolism. Plant Physiol. 2016:172(3):1371–1392. 10.1104/pp.16.00767 27609861 PMC5100799

[kiaf344-B39] Santelia D, Lunn JE. Transitory starch metabolism in guard cells: unique features for a unique function. Plant Physiol. 2017:174(2):539–549. 10.1104/pp.17.00211 28292855 PMC5462065

[kiaf344-B40] Santelia D, Trost P, Sparla F. New insights into redox control of starch degradation. Curr Opin Plant Biol. 2015:25:1–9. 10.1016/j.pbi.2015.04.003 25899330

[kiaf344-B41] Schneider CA, Rasband WS, Eliceiri KW. NIH image to ImageJ: 25 years of image analysis. Nat Methods. 2012:9(7): 671–675. 10.1038/nmeth.2089 22930834 PMC5554542

[kiaf344-B42] Schwarzländer M, Dick TP, Meyer AJ, Morgan B. Dissecting redox biology using fluorescent protein sensors. Antioxid Redox Signal. 2016:24(13): 680–712. 10.1089/ars.2015.6266 25867539

[kiaf344-B43] Schwarzländer M, Fricker MD, Müller C, Marty L, Brach T, Novak J, Sweetlove LJ, Hell R, Meyer AJ. Confocal imaging of glutathione redox potential in living plant cells. J Microsc. 2008:24(13):299–316. 10.1111/j.1365-2818.2008.02030.x 18778428

[kiaf344-B44] Seung D, Thalmann M, Sparla F, Hachem MA, Lee SK, Issakidis-Bourguet E, Svensson B, Zeeman SC, Santelia D. *Arabidopsis thaliana* AMY3 is a unique redox-regulated chloroplastic α-amylase. J Biol Chem. 2013:288(47):33620–33633. 10.1074/jbc.M113.514794 24089528 PMC3837109

[kiaf344-B45] Skryhan K, Gurrieri L, Sparla F, Trost P, Blennow A. Redox regulation of starch metabolism. Front Plant Sci. 2018:9:1344. 10.3389/fpls.2018.01344 30298078 PMC6160744

[kiaf344-B46] Souza PVL, Lima-Melo Y, Carvalho FE, Reichheld JP, Fernie AR, Silveira JAG, Daloso DM. Function and compensatory mechanisms among the components of the chloroplastic redox network. CRC Crit Rev Plant Sci. 2019:38(1):1–28. 10.1080/07352689.2018.1528409

[kiaf344-B47] Sparla F, Costa A, Lo Schiavo F, Pupillo P, Trost P. Redox regulation of a novel plastid-targeted β-amylase of Arabidopsis. Plant Physiol. 2006:141(3):840–850. 10.1104/pp.106.079186 16698902 PMC1489908

[kiaf344-B48] Storm AR, Kohler MR, Berndsen CE, Monroe JD. Glutathionylation inhibits the catalytic activity of Arabidopsis β-Amylase3 but not that of paralog β-amylase1. Biochemistry. 2018:57(5):711–721. 10.1021/acs.biochem.7b01274 29309132

[kiaf344-B49] Valerio C, Costa A, Marri L, Issakidis-Bourguet E, Pupillo P, Trost P, Sparla F. Thioredoxin-regulated β-amylase (BAM1) triggers diurnal starch degradation in guard cells, and in mesophyll cells under osmotic stress. J Exp Bot. 2011:62(2):545–555. 10.1093/jxb/erq288 20876336 PMC3003804

[kiaf344-B50] Wilkins MR, Gasteiger E, Bairoch A, Sanchez J-C, Williams KL, Appel RD, Hochstrasser DF. Protein identification and analysis tools in the ExPASy server. Methods Mol Biol. 1999:112:531–552. 10.1385/1-59259-584-7:531 10027275

[kiaf344-B51] Yoshida K, Hisabori T. Current insights into the redox regulation network in plant chloroplasts. Plant Cell Physiol. 2023:64(7):704–715. 10.1093/pcp/pcad049 37225393 PMC10351500

[kiaf344-B52] Yu J, Li Y, Qin Z, Guo S, Li Y, Miao Y, Song C, Chen S, Dai S. Plant chloroplast stress response: insights from thiol redox proteomics. Antioxid Redox Signal. 2020:33(1):35–57. 10.1089/ars.2019.7823 31989831

[kiaf344-B53] Yu X, Pasternak T, Eiblmeier M, Ditengou F, Kochersperger P, Sun J, Wang H, Rennenberg H, Teale W, Paponov I, et al Plastid-localized glutathione reductase2–regulated glutathione redox status is essential for Arabidopsis root apical meristem maintenance. Plant Cell. 2013:25(11):4451–4468. 10.1105/tpc.113.117028 24249834 PMC3875729

[kiaf344-B54] Zaffagnini M, Bedhomme M, Lemaire SD, Trost P. The emerging roles of protein glutathionylation in chloroplasts. Plant Sci. 2012a:185–186:86–96. 10.1016/j.plantsci.2012.01.005 22325869

[kiaf344-B55] Zaffagnini M, Bedhomme M, Marchand CH, Couturier J, Gao XH, Rouhier N, Trost P, Lemaire SD. Glutaredoxin S12: unique properties for redox signaling. Antioxid Redox Signal. 2012b:16(1):17–32. 10.1089/ars.2011.3933 21707412

[kiaf344-B56] Zaffagnini M, Fermani S, Marchand CH, Costa A, Sparla F, Rouhier N, Geigenberger P, Lemaire SD, Trost P. Redox homeostasis in photosynthetic organisms: novel and established thiol-based molecular mechanisms. Antioxid Redox Signal. 2019:31(3):155–210. 10.1089/ars.2018.7617 30499304

[kiaf344-B57] Zanella M, Borghi GL, Pirone C, Thalmann M, Pazmino D, Costa A, Santelia D, Trost P, Sparla F. β-amylase 1 (BAM1) degrades transitory starch to sustain proline biosynthesis during drought stress. J Exp Bot. 2016:67(6):1819–1826. 10.1093/jxb/erv572 26792489

[kiaf344-B58] Zimmer D, Swart C, Graf A, Arrivault S, Tillich M, Proost S, Nikoloski Z, Stitt M, Bock R, Mühlhaus T, et al Topology of the redox network during induction of photosynthesis as revealed by time-resolved proteomics in tobacco. Sci Adv. 2021:7(51):eabi8307. 10.1126/sciadv.abi8307 34919428 PMC8682995

