# Supplementary material for: Dynamic regulation of Arabidopsis β-AMYLASE1 by glutathione and thioredoxins affects starch in guard cells
Source: Plant Physiol. 2025 Aug 1;198(4):kiaf344. doi: 10.1093/plphys/kiaf344 (PMC12351283; doi:10.1093/plphys/kiaf344)
Supplement: kiaf344_Supplementary_Data [file kiaf344_supplementary_data.pdf]

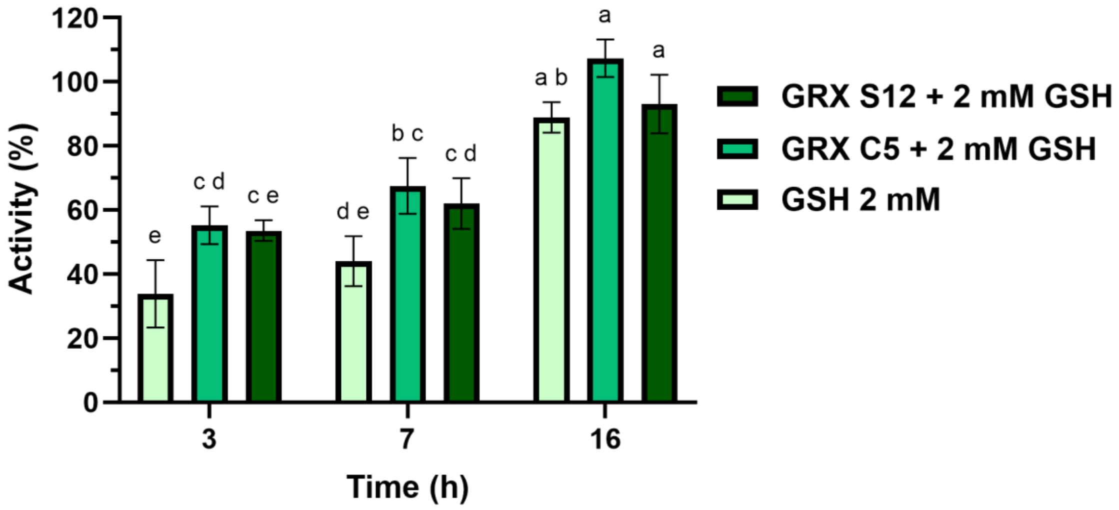

Supplementary Figure S1 - GSH- and GRX-dependent recovery of BAM1 activity requires prolonged incubation. The reversibility of inactivation of BAM1 was assessed on inhibited BAM1 samples obtained after treatment with 1 mM GSSG for 1 h, desalted and incubated for 90 minutes at 37° C. Inhibited BAM1 was incubated in presence of 2 mM GSH alone or together with 1 µM GRX C5 or GRX S12. Data were expressed as percentage of fully reduced BAM1 and analyzed with two-way ANOVA and Tukey's test with $p<0.05$, where distinct lowercase letters denote significant group differences. The experiment was carried out in triplicate, error bars show standard deviation..
